# Molecular investigation and clinical management of *Hepatozoon Canis* infection in an Indian jackal – a case report

**DOI:** 10.1186/s12917-022-03213-8

**Published:** 2022-04-20

**Authors:** S.M. Kolangath, S.V. Upadhye, V.M. Dhoot, M.D. Pawshe, A.S. Shalini, R.M. Kolangath

**Affiliations:** 1 Wildlife Research & Training Centre, Gorewada, MAFSU, Nagpur Opp. Hindustan Lever Godown Square, Mahurzhari Road, Fetri, Nagpur, 441501 India; 2 Department of Biotechnology & Biochemistry, Saint Francis DeSales College, Seminary Hills, Nagpur, 440006 India

**Keywords:** *Hepatozoon canis*, *Leucocytozoon*, Indian jackal, Doxycycline, *Rhipicephalus*, Wildlife, Conservation efforts

## Abstract

**Background:**

Hepatozoonosis is a common tick-borne illness reported from all over the world. The infection has been well documented in dogs and cats, and has also been identified in wild canids and felids. India is home to many canid species; however, the incidence of Hepatozoonosis in wild canids is rarely reported. A wide variety of protocols have been discussed for the clinical management of the infection in companion animals; however, the suitability of treatment protocols in wild canids is understudied. The current case report highlights the clinical management of Hepatozoonosis in an Indian jackal and molecular investigation to provide vital insights into the epidemiology of the disease.

**Case Presentation:**

A paraplegic Indian jackal was rescued from Melghat Tiger Reserve, Maharashtra, India. The animal had extensive decubital ulcers on the left pin bone and could not walk; however, the animal was active and dragged the hindlimb during locomotion. The vital parameters, blood and serum investigations were normal. Post physiotherapy, massage and infrared therapy, the animal could walk but started knuckling, resulting in injuries. Eight weeks into rehabilitation, the animal had a steep fall in haemoglobin concentration, platelet count, weight loss and was diagnosed with Hepatozoonosis. Considering the altered vital parameters, the jackal was rationally treated with Doxycyclin @ 20 mg/Kg O.D. (Once Daily) for 45 days along with supportive therapy. The jackal recovered after the treatment and led a normal life.

**Conclusion:**

Mono-drug regime using Doxycycline was effective in the alleviation of *H.canis* infection in jackal. The drug was effective in alleviating the clinical presentation without alteration of vital parameters. The molecular investigation provided qualitative inputs in understanding the epidemiology of *Hepatozoon* in wild canids.

## Background

Tick-borne diseases have been extensively studied in companion animals; however, the epidemiology of protozoal diseases in the wild is understudied. Ticks transmit various infections in the tropical region. The hot and humid climate of the tropics is conducive to the propagation of ticks [1]. There are numerous reports of tick-borne diseases emerging from the Indian subcontinent every year. Hepatozoonosis is a tick-borne disease of companion and wild animals. *Rhipicephalus* and *Amblyomma* species are considered important transmitting agents for infection. Recently, the role of *Dermacentor reticulatus* has been investigated in the transmission of multiple infections, including Hepatozoonosis in red fox (*Vulpes vulpes*) [2]. The infection has been reported in dogs [3] and cats [4–7]; however, few reports from the wild have been documented. The reports of wild are primarily limited to canids [8–10], ursids [11], felids [12–17], rodents [18], reptiles [19], mustelids [20] and procyonids [21]. Dogs are considered reservoirs of *H. canis*; they can be infested with brown dog ticks (*Rhipicephalus* sp.), the definitive hosts. Many species of wild felids and canids are known to be infected with Hepatozoonosis. India has reported incidences of *H. canis* in wild dogs (*Cuon alpinus*) in the recent past; however, no reports in any other wild canid species have surfaced from the region [12]. The current case report highlights the clinical management and molecular investigation of Hepatozoonosis in an Indian jackal (*Canis aureus indicus*).

## Case presentation

An adult male Indian jackal (*Canis aureus indicus*) was rescued from Melghat Tiger Reserve, Maharashtra, India with a history of paraplegia due to a possible automobile accident. The animal was rationally treated at the Transit Treatment Centre and was transferred to Wildlife Research & Training Centre, Gorewada, Nagpur, for further care and rehabilitation after a couple of months. On reception the vital parameters, blood and serum values were within the normal range. The radiological examination revealed an L6 spinal fracture with L6-L7 dislocation resulting in paraplegia. The animal was treated rationally with Injection Ceftiofur Sodium @ 22 mg/Kg, Injection Methyl Prednisolone 2.2 mg/ Kg, Injection Ranitidine HCl (Hydrochloride) @ 4.4 mg total dose once a day. This was supplemented with physiotherapy and infra-red therapy. Post 15 days into rehabilitation, the jackal was found to support his weight on the hind limbs and could walk. However, it was noticed that the jackal started knuckling during the walk and resulted in lacerations and wounds.

**Fig. 1:**
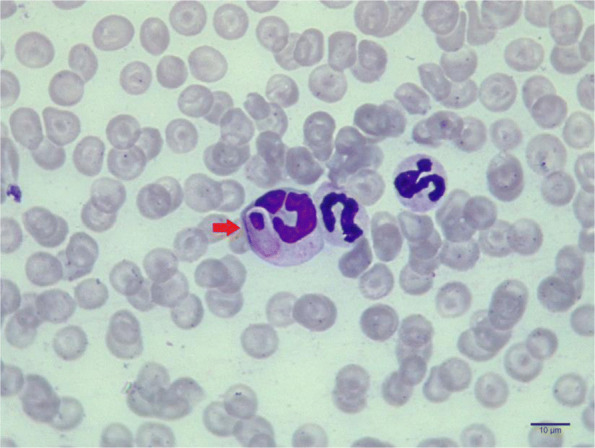
Blood smear examination revealed presence of gamonts in the neutrophils (indicated by arrow). Normal neutrophils can be seen in the periphery

The jackal exhibited fever, anaemia, depression, weight loss, rough hair coat, lethargy and inappetence on day 55 of rehabilitation (Day 0). Blood investigation revealed decreased haemoglobin concentration (6.1 g/dL), thrombocytopenia, leucocytosis and neutrophilia; peripheral blood smear examination revealed anisocytosis and the presence of gamonts in neutrophils (Fig. 1). Serum biochemistry showed hypoglycemia and elevated AST (Aspartate Aminotransferase). On day 0, 12.5% of the circulating neutrophils were found to contain gamonts. Considering the low haemoglobin concentration and altered vitals, a mono antiprotozoal therapy was employed using Doxycycline @ 20 mg/Kg body weight once a day along with supportive therapy including hepatoprotectants, iron supplements, vitamins and probiotics. The mono antiprotozoal regime was continued for 45 days, during which vital parameters were monitored on days 0, 21, 45 (Table 1) and monthly thereafter. During the course of the therapy, blood indices and clinical presentation gradually improved, and the percentage of neutrophils containing gamonts gradually improved, and the percentage of neutrophils containing gamonts gradually tapered from 12.5%, 7.66% to 0% on days 0, 21 and 45, respectively.

**Table 1:** Haemato-biochemical values and peripheral blood smear studies evaluated on Day 0, 21 and 45 along with normal reference values

Sr. No.	Parameter	On Arrival	Day 0	Day 21	Day 45	Reference Value
1	Lymphocytes (%)	14.2	16.5	16	18	12-30
2	Monocytes (%)	6.4	8.2	6	5.2	3-10
3	Neutrophils (%)	76.2	74.7	76	72.7	60-70
4	Eosinophils (%)	3.0	0.4	2	3.8	2-10
5	Basophils (%)	0.2	0.2	00	0.3	0-1
6	WBC (10^9^/L)	19.87	24.87	20.43	18.32	6-17
7	RBC (10^12^/L)	7.14	6.92	6.08	7.01	5.5-8.5
	Platelets (10^9^/L)	419	192	212	321	200-500
8	Haemoglobin (g/dL)	11.65	6.1	6.8	7.4	12-18
9	HCT	37.65	37.16	38.02	38.23	37-55
10	Mean Corpuscular Volume (fL)	59	54	55	57	58-79
11	Mean Concentration Haemoglobin (MCH) (pg)	16.8	16.0	16.9	16.79	19.5-24.5
12	MCHC (g/dl)	31.2	29.7	30.4	30.8	32-36
13	BUN (mg/dL)	21	20	20	29	7-25
14	Creatinine (mg/dL)	0.6	0.6	0.5	0.6	0.3-1.4
15	ALT (U/L)	52	58	100	48	10-118
16	AST (U/L)	58	61	62	51	14-45
17	ALP (U/L)	68	71	26	36	20-150
18	Total Bilirubin (mg/dl)	0.3	0.3	0.3	0.3	0.1-0.6
19	Glucose (mg/dl)	72	31	88	76	60-110
20	Calcium (mg/dl)	8.6	8.6	9.1	9.0	8.6-11.8
21	Total Protein (g/dL)	5.8	6.0	6.6	6.9	5.4-8.2
22	Albumin (g/dL)	2.4	2.5	2.6	2.6	2.5-4.4
23	Globulin (g/dL)	3.4	3.5	4.0	4.3	2.3-5.2
24	Na^+^ (mmol/ L)	138	136	138	138	138-160
25	K^+^ (mmol/ L)	5.4	5.4	5.2	4.8	3.7-5.8
26	Cl- (mmol/ L)	105	105	105	116	95-119
27	tCO_2_ (mmol/ L)	19	19	22	18	12-27
28	Body weight (Kg)	6.7	6.3	6.1	6.63	
29	Peripheral Smear	Normal	Hepatozoon +++ Thrombocytopenia	Hepatozoon +	Normal	
30	% of neutrophils containing gamont	None	12.5%	07.66%	None	
31	Clinical Presentation	Normal appetite; active, wound on pin bone, urine & faeces normal, locomotion by dragging hind limbs, drag injuries. Vital parameter normal	Fever, depression, inappetence, lethargy, urine & faeces normal, weight loss, anaemia	Lethargy, faeces and urine normal, appetite slightly reduced, anaemia	Active, wounds healed, appetite restored, active, weight gained, anaemia	

Reference: Haematology and Serum Biochemistry reference ranges, 10th edn. The Merck

Veterinary Manual (Cyntia, 2011)

**Fig. 2:**
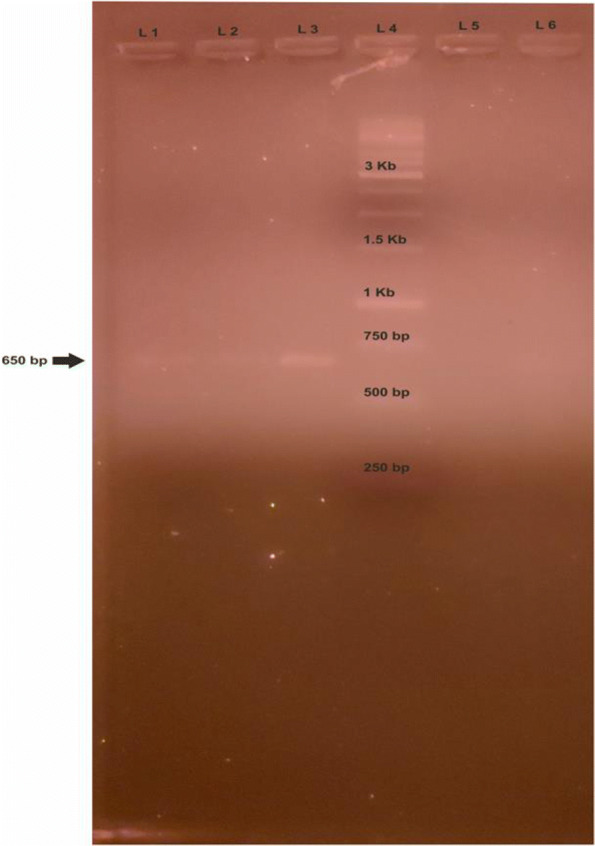
Gel electrophoresis on 1% agarose gel. Lane 1,2 and 3 PCR reaction performed on days 35, 21 and 0 respectively, L4 1 Kb ladder, L5 Day 45 and L6 Negative control. An amplification of 650 bp obtained using HEP-F and HEP-R primer (Inokuma et al., 2002)

**Fig. 3:**
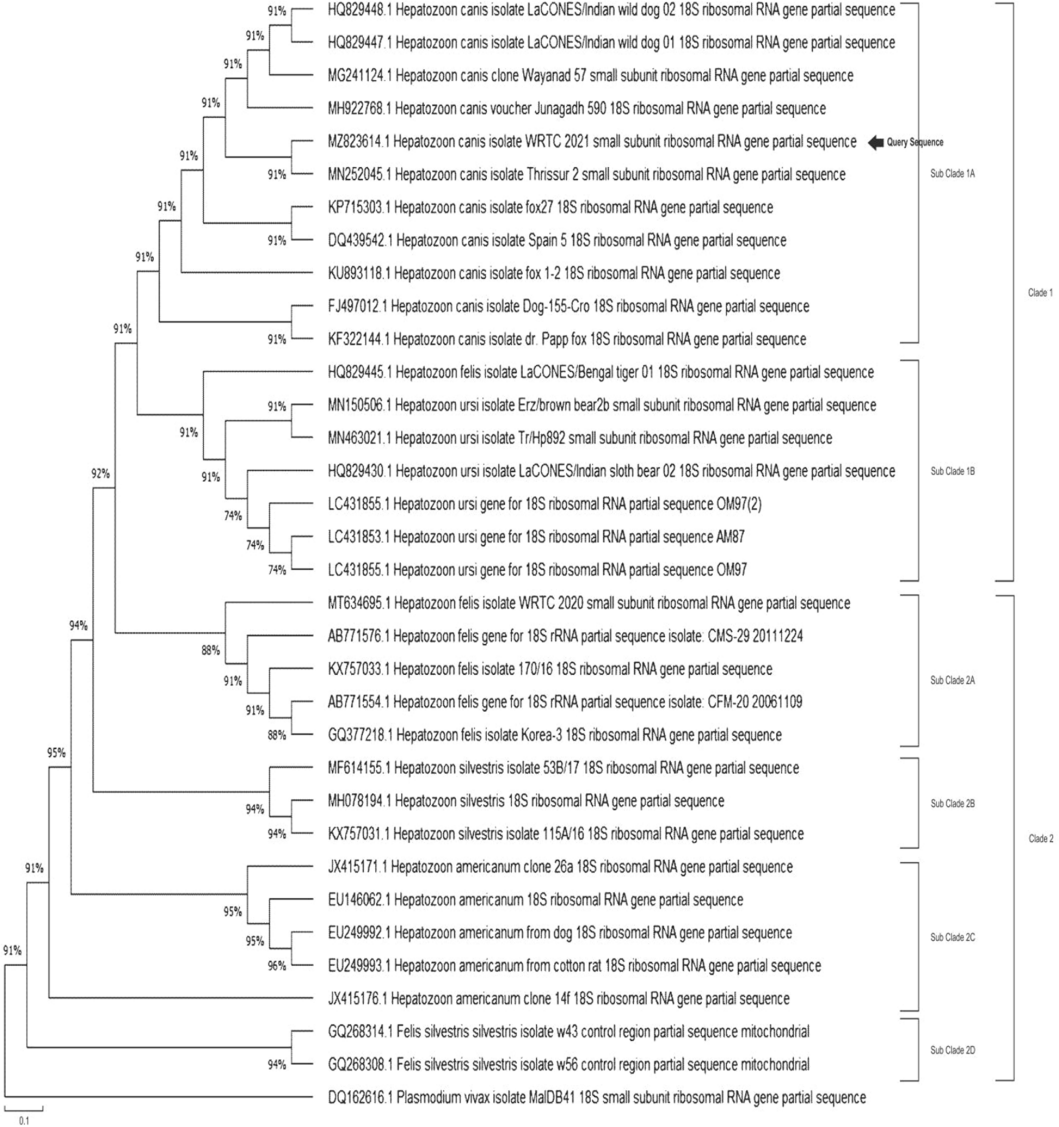
Phylogenetic Analysis of Sequence by Neighbour Joining Phylogenetic Tree using Bootstrap Method (1000 replications)

To further investigate the infection, DNA (Deoxyribonucleic Acid) was isolated from 100 *μ*l of whole blood using DNeasy$\circledR $ Blood and Tissue Kit (Mfg. Qiagen Inc, MD, USA) as per the manufacturer’s instructions and Polymerase Chain Reaction (PCR) was performed targeting the 18S ribosomal RNA gene of *Hepatozoon* sp [22]. An amplification of 650 bp was obtained (Fig. 2), which was sequenced using HepF and HepR primer pairs on ABI 3130 automated DNA sequencer (Mfg. Applied Biosystems, CA, USA). *H. felis* amplicon reported from tiger was used as a positive control, while the negative control was devoid of any template. To monitor the progression of the infection, PCR studies were repeated on days 21 and 35 (Fig. 2, Lane 1, 2 and 3). To limit relapses, blood samples were examined every month and were found to be negative consistently. The obtained sequence was submitted to the NCBI nucleotide database using the nBLAST tool. It was found to be 99.68% identical to the previously reported *H. canis* in dogs from India (Accession No. MH922768, MN252045). A systematic phylogenetic analysis was undertaken by including sequences of *Hepatozoon* identified in wild canids, ursids and felids. The sequences were selected based on similarity with the query sequence (Details in Table2). Sequences reported in domestic and wild were included without preference. Neighbour joining phylogenetic trees using the bootstrap method were constructed using Mega X software to study the topologies of the phylogenetic trees (Fig. 3) [23]. The bootstrap values (1000 replications) were analysed to ensure tree consistency.

**Table 2:** List of sequences along with their attributes used for the neighbour-joining phylogenetic studies

Sr. No.	Accession No.	Organism	Host	Country	Author
1	HQ829448	Hepatozoon canis	Cuon alpinus	India	[12]
2	HQ829447	Hepatozoon canis	Cuon alpinus	India	[12]
3	KF322144	Hepatozoon canis	Vulpes vulpes	Hungary	[38]
4	KU893118	Hepatozoon canis	Vulpes vulpes	Czech Republic	[39]
5	FJ497012	Hepatozoon canis	Canis lupus fimilaris	Croatia	Vajota et al., 2009 (Unpublished)
6	KP715303	Hepatozoon canis	Vulpes vulpes	Italy	[40]
7	DQ439542	Hepatozoon canis	Vulpes vulpes	Spain	Criado-Fonelio., 2007
8	MZ823614	Hepatozoon canis	Canis aureus indicus	India	Kolangath et al., 2021 (Unpublished)
9	MN252045	Hepatozoon canis	Canis lupus fimilaris	India	Angeline et al., 2019 (Unpublished)
10	MH922768	Hepatozoon canis	Canis lupus fimilaris	India	[19]
11	MG241124	Hepatozoon canis	Hemaphysalis	India	[41]
12	MN150506	Hepatozoon ursi	Ursus arctos	Turkey	[42]
13	MN463021	Hepatozoon ursi	Ursus arctos	Turkey	[43]
14	LC431855	Hepatozoon ursi	Ursus thibetanus japonicus	Japan	[44]
15	LC431853	Hepatozoon ursi	Ursus thibetanus japonicus	Japan	[44]
16	HQ829430	Hepatozoon ursi	Melursus ursinus	India	[11]
17	LC431855	Hepatozoon ursi	Ursus thibetanus japonicus	Japan	[44]
18	HQ829445	Hepatozoon felis	Panthera tigris tigris	India	[12]
19	MT634695	Hepatozoon felis	Panthera tigris tigris	India	Kolangath et al., 2020 (Unpublished)
20	GQ377218	Hepatozoon felis	Prionailurus bengalensis	Korea	[17]
21	KX757033	Hepatozoon felis	Felis silvestris silvestris	Bosnia & Herzegovina	[45]
22	AB771554	Hepatozoon felis	Prionailurus bengalensis eupilurus	Japan	[46]
23	AB771576	Hepatozoon felis	Prionailurus bengalensis eupilurus	Japan	[46]
24	MH078194	Hepatozoon silvestris	Felis catus	Switzerland	[47]
25	KX757031	Hepatozoon silvestris	Felis silvestris silvestris	Bosnia & Herzegovina	[48]
26	MF614155	Hepatozoon silvestris	Felis silvestris silvestris	Bosnia & Herzegovina	[45]
27	GQ268314	Hepatozoon silvestris	Felis silvestris silvestris	Germany	Hertwig et al., 2009 (Unpublished)
28	GQ268314	Hepatozoon silvestris	Felis silvestris silvestris	Germany	Hertwig et al., 2009 (Unpublished)
29	JX415171	Hepatozoon americanum	Canis latrans	USA	[49]
30	EU146062	Hepatozoon americanum	Canis lupus fimilaris	USA	[50]
31	EU249992	Hepatozoon americanum	Canis lupus fimilaris	USA	[50]
32	EU249993	Hepatozoon americanum	Sigmodon hispidus	USA	[50]
33	JX415176	Hepatozoon americanum	Canis latrans	USA	[49]
34	DQ162616	*Plasmodium vivax* (Outgroup)	——-	Brasil	[51]

## Discussion and conclusion

Most of Hepatozoon spp. infection in wild and companion animals is subclinical, and it produces clinical outcomes only in very young and immunocompromised individuals [10]. Reports of death in hyenas [8] and coyotes [24] due to Hepatozoonosis highlight the need to investigate the disease among endangered wild canids. The impact of stress, habitat destruction and concurrent viral and bacterial infection on the spread of Hepatozoonosis under free-range has been studied [25]. Conventionally the diagnosis of Hepatozoonosis is primarily based on the blood smear examination; however, when the quantum of infection is scarce, the detection of infection becomes uncertain. Also, Rhipicephalus spp. transmit an array of protozoal infections, coinfection of *Anaplasma platys*, *Ehrlichia canis*, *H. canis* and *Rickettsia monacensis* has been reported in dogs [26] and wild canids [2, 27]. Molecular techniques can provide sensitive diagnostic tools for detecting protozoal diseases. When augmented with molecular tools like sequencing, qualitative inputs on the epidemiological aspects can be provided.

In the current case report, the jackal exhibited a steep fall in haemoglobin concentration, neutrophilia, leucocytosis, elevated AST levels indicative of hepatic involvement [28, 29]. During the treatment, blood parameters and corpuscular indices showed marginal improvement, with the haemoglobin concentration improving slightly from 6.1 gm/dl (Day 0) to 7.4 gm/dl (Day 45). Clinically, the animal regained its weight and began having a normal appetite and behaviour. The animal showed two instances of loose stools during treatment which was rationally treated with probiotics and diet management. The haemoglobin concentration returned to 10.5 after 90 days, probably due to concurrent blood loss from nursing injuries. Relapses of Hepatozoonosis have been reported in dogs [30, 31]. Hence, a monthly peripheral blood smear and PCR tests were conducted to rule out the relapse, which was consistently negative.

The selection of a therapeutic agent is critical in treating infection in patients with altered vital parameters. The therapeutic agent for treating Hepatozoonosis is selected based on the species of *hepatozoon* identified. The drugs employed vary from agent to agent; for *H. americanum*, a multi-drug combination of trimethoprim, pyrimethamine, clindamycin, decoquinate [32]. Also, anticoccidial agent toltrazuril has been found effective in the treatment of experimentally infected voles with *H. erhardovae* [33]. However, *H. canis* responds to a combination of imidocarb dipropionate and Doxycycline [29, 34]. Side-effects like hepatic necrosis, kidney failure and death have been reported in dogs treated with imidocarb [35], and the drug has failed to eliminate *H. canis* in naturally infected dogs [36]. Considering the safety and duration of treatment, mono drug therapy of Doxycycline for 45 days was opted for. The elimination of *Hepatozoon* infection on day 45 was confirmed by blood smear examination and PCR (Fig. 2 lane 5). It is worth pointing out that the jackal showed no deviations from normal during blood and serum examination on reception. The upsurge of infection was in coincidence with trauma and stress due to tissue injury, as reported in Lions [37]. The jackal recovered uneventfully; thus, Doxycycline has value in the clinical management of *H. canis* infection in wild canids with compromised vitals.

A Polymerase Chain Reaction (PCR) was carried out to confirm the finding further [22]. The amplicon of 650 bp was sequenced and submitted to National Center for Biotechnology Information (NCBI) database for reference. The sequence was allotted accession number MZ823614. In the current study, sequence analysis revealed 99.68% identity with previously reported sequences of *H. canis* in dogs from India (Accession No. MH922768, MN252045). There are only a couple of previously reported *H. canis* in wild dogs (*Cuon alpinus*) from India (Accession No. HQ829448, HQ829447). No reports of Hepatozoonosis from other wild species have been reported from the country. The phylogenetic analysis revealed the presence of the query sequence (MZ823614) in clade I A consisting of sequences of *H. canis* reported in dogs and other wildlife from India and abroad. The major clade II consisted mainly of the sequences of *H. felis* reported in wild felids from India, Korea, Bosnia & Herzegovina (Table2); *H.silvestris* and *H. americanum* isolated from a variety of host species including wild and domestic animals (Details provided in Table2). *Plasmodium vivax* formed a distinct outgroup.

The case report highlights the utility of oral Doxycycline in the clinical management of Hepatozoonosis in wild canids with altered vitals. Molecular techniques are sensitive and can be effectively utilised in the monitoring of the progression of the disease. The insights provided by high throughput technologies like sequencing can help understand the epidemiology and prevalence of Hepatozoonosis. It is essential to study the dynamics of the infection in the wild to ensure the survival of the endangered wildlife. Further investigation in sympatric canids species in the wild and captivity can provide critical insights on Hepatozoonosis.

## Data Availability

The sequence identified in the study is available in the public domain database of nCBI under accession no. mZ823614. https://www.ncbi.nlm.nih.gov/nuccore/MZ823614

## Abbreviations

O.D.: Once Daily
HCl: Hydrochloride
PCR: Polymerase Chain Reaction
DNA: Deoxyribonucleic Acid
AST: Aspartate Aminotransferase
NCBI: National Center for Biotechnology Information
